# Association between tobacco smoke exposure and serum parathyroid hormone levels among US adults (NHANES 2003–2006)

**DOI:** 10.1038/s41598-024-66937-7

**Published:** 2024-07-09

**Authors:** Longqing Hu, Bei Qian, Kaijian Bing, Li Mei, Shengnan Ruan, Xincai Qu

**Affiliations:** Department of Thyroid and Breast Surgery, Union Hospital, Tongji Medical College, Huazhong University of Science and Technology, 1277 Jiefang Avenue, Wuhan, 430022 China

**Keywords:** Environmental impact, Disease prevention

## Abstract

Tobacco smoke exposure has been demonstrated to impede bone remodeling and diminish bone density, yet research regarding its correlation with parathyroid hormone (PTH) remains limited. This study aims to investigate the relationship between tobacco smoke exposure and serum PTH levels in adults aged 20 years and older. This study included 7,641 participants from two cycles of the National Health and Nutrition Examination Survey (NHANES, United States, 2003- 2006). Reflect tobacco smoke exposure through serum cotinine levels, and use an adjusted weighted multivariate linear regression model to test the independent linear relationship between serum cotinine and PTH. Stratified analysis was conducted to validate the sensitivity of the conclusions. Smooth curve fitting and threshold effect analysis were performed to assess the non-linear relationship. After comprehensive adjustment using weighted multivariate regression analysis, a negative correlation was found between serum cotinine and PTH levels. The interaction p-values in subgroup analyses were all greater than 0.05. Moreover, smooth curve fitting indicated a non-linear relationship between serum cotinine and PTH, with a turning point observed. Our research indicates that tobacco smoke exposure is negatively correlated and independent of serum parathyroid hormone levels, indicating that long-term tobacco smoke exposure may lead to parathyroid dysfunction in adults.

## Introduction

Parathyroid hormone (PTH) is an alkaline single-chain polypeptide hormone secreted by the chief cells of the parathyroid glands, playing an important role in regulating the calcium-phosphate balance in the body^1^. PTH can act on the skeleton, kidneys, and other organs, regulating bone remodeling and synthesis, as well as controlling the kidneys’ reabsorption of calcium and phosphate salts^2^. The PTH levels in the human body are regulated by blood calcium levels and are also influenced by other various factors such as body weight, renal function, and vitamin D levels^3,4^.

Tobacco smoke exposure poses a significant challenge to human health. Both active smoking and exposure to secondhand smoke (SHS) at home and in the workplace can lead to tobacco smoke exposure^5^. Commercial cigarettes release various harmful substances during combustion, including tar, nicotine, and nitrosamines^6^. Tobacco smoke exposure has been confirmed to be associated with various diseases, including thyroid dysfunction, osteoporosis, and several types of cancers^7–9^. Due to the short half-life and instability of nicotine in the human body, it is challenging to use it as an indicator to assess smoke exposure^10^. Cotinine is the primary metabolite of nicotine formed by the body's metabolism of tobacco combustion products. Due to its long half-life and ease of metabolic elimination from the human body, the serum levels of cotinine can reflect the extent of exposure to tobacco smoke. Consequently, it is frequently employed as a biomarker for assessing tobacco smoke exposure in biological specimens within scholarly research^11^.

Clinical epidemiological research has confirmed that smoking disrupts bone homeostasis, serving as an independent risk factor for osteoporosis^12^. Its mechanism may be achieved through inducing iron-mediated apoptosis in bone marrow mesenchymal stem cells^13^. Although PTH plays a crucial role in maintaining bone homeostasis and calcium-phosphate balance, existing studies have primarily focused on the relationship between smoking and osteoporosis, often limited to specific populations^14,15^. However, there is still a lack of direct evidence regarding the association between tobacco smoke exposure and PTH levels. This study aims to explore the relationship between serum cotinine levels and PTH, extending the findings to a broader population to gain a deeper understanding of the impact of tobacco smoke exposure on PTH levels. This will offer new insights and more effective strategies for the prevention and management of osteoporosis and other bone health issues.

## Methods

### Data source

The National Health and Nutrition Examination Survey (NHANES) is conducted by the National Center for Health Statistics (NCHS) and the Centers for Disease Control and Prevention (CDC). NHANES selects participants through biennial, multi-stage random sampling and assesses the overall health and nutritional status of the U.S. population through examinations and interviews. All open data used in this study are sourced from the official NHANES website (https://www.cdc.gov/nchs/nhanes/index.htm), which is updated every two years.

### Study population

**Figure 1:**
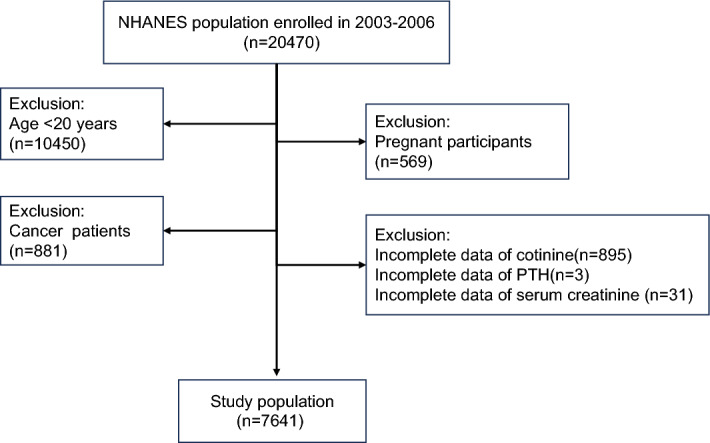
Participant inclusion flowchart.

Participants received standardized family interviews and underwent health checks at mobile examination centers, collecting laboratory data through various tests to evaluate their medical and physiological conditions. Due to the variability in laboratory examination items within the NHANES database, we conducted statistical analysis on the data. Ultimately, we selected two NHANES cycles (2003–2004 and 2005–2006) during which both serum cotinine and parathyroid hormone data could be simultaneously obtained. Initially, the study included 20,470 participants. Participants were excluded from our study based on the following criteria: (1) age < 20 years (n = 10,450), (2) pregnancy (n = 569), (3) cancer patients (n = 881), (4) missing complete information on Cotinine (n = 895), PTH (n = 3), and serum creatinine (n = 31). As shown in Fig. 1, our study ultimately included 7,641 eligible participants.

### Cotinine and PTH measurement

Serum cotinine levels were measured using the isotope dilution-high-performance liquid chromatography/atmospheric pressure chemical ionization tandem mass spectrometry (ID HPLC-APCI MS/MS) method. The Elecsys 1010 method was used for in vitro quantitative determination of intact parathyroid hormone in human serum and plasma. The measurement methods and locations remained unchanged across these two NHANES cycles.

### Covariates

In this study, we used serum PTH as the outcome variable related to serum cotinine. Serum PTH levels are influenced by various factors. Based on previous related articles, we selected the following covariates to enhance the robustness of the association^16–18^. The categorical variables included gender, race/ethnicity, education level (Lower than high school/High school/College or above), diabetes history (Yes/No/Borderline), hypertension history (Yes/No), alcohol consumption (Yes/No) and smoke (Yes/No). Information on diabetes history, hypertension history, and alcohol consumption was obtained through questionnaires. Alcohol history was defined as consuming more than 12 units of alcohol in the past year. Smoking is defined as having smoked at least 100 cigarettes in life.

The continuous variables included age (years), family poverty income ratio (PIR), body mass index (BMI) (kg/m^2^), 25 (OH) D_3_ (nmol/mL), serum albumin (g/dL), serum creatinine (mg/dL), alkaline phosphatase (U/L), serum calcium (mg/dL), cholesterol (mg/dL), triglycerides (mg/dL), serum glucose (mg/dL), phosphorus (mg/dL), urinary albumin (ug/mL), and urinary creatinine (mg/dL).

Serum corrected calcium was calculated using serum albumin and serum calcium. When serum albumin levels were < 4.0 g/dL, the following formula was used to correct serum calcium levels ^19^: Corrected calcium (mg/dL) = measured total calcium (mg/dL) + 0.8 × [4.0 - serum albumin (g/dL)]. The CKD-EPI equation was used to calculate the estimated glomerular filtration rate (eGFR, ml/min/1.73 m^2^)^20^, with eGFR below 60 ml/min/1.73 m^2^ defining chronic kidney disease (CKD).

### Statistical analysis and sensitivity analysis

NHANES sampling weights were constructed for merging survey cycles statistical analysis. We used the WTMEC2YR weighting to analyze the data. Categorical variables were presented as percentages, normally distributed continuous variables were expressed as mean (SD), and non-normally distributed continuous variables were presented as median (Q1, Q3). Chi-square tests were employed for inter-group distributions of categorical variables, while the Kruskal–Wallis test was used for non-normally distributed continuous variables.

To analyze the sensitivity of the study, due to the severely right-skewed distribution of cotinine data, we applied the natural logarithm (LN) transformation to cotinine and utilized it in regression analysis and stratified analysis. At the same time, we performed multiple imputations for missing data to avoid bias. Five imputed datasets were created using the “MICE” package with a chained equation approach. Finally, generalized additive models (GAM) were used to construct smooth curve fitting to determine the nonlinear relationship between cotinine and PTH. Additionally, threshold effect analysis models were used to detect the inflection point for the relationship between cotinine and PTH.

All analysis was performed using Empower software (www.empowerstats.com; X&Y solutions, Inc., Boston MA) and R software (version 4.2.2; http://www.R-project.org, R Foundation for Statistical Computing, Vienna, Austria). P values less than 0.05 were considered statistically significant.

### Ethical approval

All open data used in this study are sourced from the official NHANES website (https://www.cdc.gov/nchs/nhanes/index.htm). Institutional Review Board (IRB) and NCHS Research Ethics Review Board (ERB) have granted research authorization, eliminating the need for additional permits.

## Results

### Basic characteristics of included participants

A total of 7641 participants were included in this study. Weighted baseline characteristics were stratified by PTH quartiles and are presented in Supplementary Table S1. In our study, participants were predominantly non-Hispanic white. Participants with high PTH levels are more likely to have lower family income and lower education levels, a higher incidence rate of hypertension, and lower rates of alcohol consumption and smoking. Meanwhile, participants with high levels of PTH had lower serum albumin levels, 25 (OH) D_3_ levels, serum corrected calcium levels, serum phosphorus levels, and eGFR levels, while higher BMI, alkaline phosphatase levels, serum glucose levels, urinary albumin and creatinine levels.

### Relationship between serum cotinine and PTH

We constructed three weighted linear regression models for cotinine and PTH (Table 1), each incorporating different covariates to verify the reliability and sensitivity of the association. We found that there was a statistically significant negative correlation between Ln-cotinine and PTH in all models. In Model 3, this negative correlation remains stable, indicating that for every unit increase in Ln-cotinine, PTH decreases by 0.58 pg/mL (0.72, 0.45, p < 0.001).

**Table 1:** Multivariate weighted linear models for the relationship between serum cotinine and PTH.

Ln-cotinine(ng/dL)	Model 1	Model 2	Model 3
Continuous	−0.85 (−0.99, −0.70) < 0.001*	−0.61 (−0.76, −0.47) < 0.001*	−0.58 (−0.72, −0.45) < 0.001*
Categories			
Quartile 1	Reference	Reference	Reference
Quartile 2	0.00 (−1.65, 1.65) 0.9961	0.16 (−1.46, 1.77) 0.8488	0.12 (−1.31, 1.55) 0.8701
Quartile 3	−1.53 (−3.20, 0.15) 0.0744	−0.57 (−2.24, 1.10) 0.5060	−1.11 (−2.62, 0.40) 0.1485
Quartile 4	−7.57 (−9.19, −5.95) < 0.001*	−5.26 (−6.90, −3.63) < 0.001*	−5.28 (−6.81, −3.76) < 0.001*

Model 1: No adjustment; Model 2: Adjustments based on age, gender, and race; Model 3: Adjust according to age, gender, race, BMI, family PIR, education, diabetes, hypertension, alcohol use, 25 (OH) D_3_, serum albumin, serum ALP, serum corrected calcium, cholesterol, triglyceride, serum creatinine, eGFR, serum glucose, serum phosphorus, urinary albumin, and urinary creatinine. Perform logarithmic conversion on cotinine.

^*^p < 0.05.

In addition, we conducted sensitivity analysis by converting Ln-cotinine from a continuous variable to a categorical variable (quartile) and calculated the trend P value (Table 1), which further confirms the negative correlation between serum cotinine and PTH.

### Stratified analysis of the relationship between serum cotinine and PTH

In Table 2, we conducted additional analysis on the relationship between Ln-cotinine and PTH in specific subgroups, stratified by age, gender, BMI, eGFR, alcohol consumption, diabetes, hypertension and other factors. In the subgroup with eGFR < 60 ml/min/1.73 m^2^, there was no statistically significant association between cotinine and PTH. In the subgroup with eGFR ≥ 60 ml/min/1.73 m^2^, for each unit of increase in Ln-cotinine, PTH decreased by 0.58 pg/mL (0.72, 0.44, p < 0.001). In all subgroups, the interaction p-value was greater than 0.05, proving a negative correlation between serum cotinine and PTH.

**Table 2:** Stratified analysis of the relationship between serum cotinine and PTH.

Subgroup	β (95% CI)	P value	P for interaction
Age (year)			0.4256
< 47	−0.60 (−0.78, −0.43)	< 0.001	
≥ 47	−0.71 (−0.92, −0.50)	< 0.001	
Gender			0.2865
Male	−-0.52 (−0.70, −0.34)	< 0.001	
Female	−0.66 (−0.86, −0.46)	< 0.001	
BMI (kg/m^2^)			0.8848
< 25	−0.57 (−0.79, −0.35)	< 0.001	
25–30	−0.65 (−0.87, −0.42)	< 0.001	
> 30	−0.60 (−0.83, −0.36)	< 0.001	
eGFR (ml/min/1.73 m^2^)			0.1861
< 60	−0.16 (−0.77, 0.45)	0.5979	
≥ 60	−0.58 (−0.72, −0.44)	< 0.001	
Drink			0.7892
Yes	−0.57 (−0.73, −0.42)	< 0.001	
No	−0.62 (−0.90, −0.34)		
Diabetes			0.0710
Yes	−0.07 (−0.59, 0.44) 0.7791	0.2188	
No	−0.61 (−0.75, −0.46)	< 0.001	
Edge	−1.25 (−2.36, −0.13) 0.0284	0.0245	
Hypertension			0.6875
Yes	−0.63 (−0.88, −0.38)	< 0.001	
No	−0.57 (−0.73, −0.41)	< 0.001	

Age, gender, race, BMI, family PIR, education, diabetes, hypertension, alcohol consumption, 25 (OH) D_3_, serum albumin, serum ALP, serum corrected calcium, cholesterol, triglycerides, serum creatinine, eGFR, serum glucose, serum phosphorus, urinary albumin, and urinary creatinine were adjusted (except for subgroup factors). Perform logarithmic conversion on cotinine.

^*^p < 0.05.

### Analysis of nonlinear and threshold effects between serum cotinine and PTH

**Figure 2:**
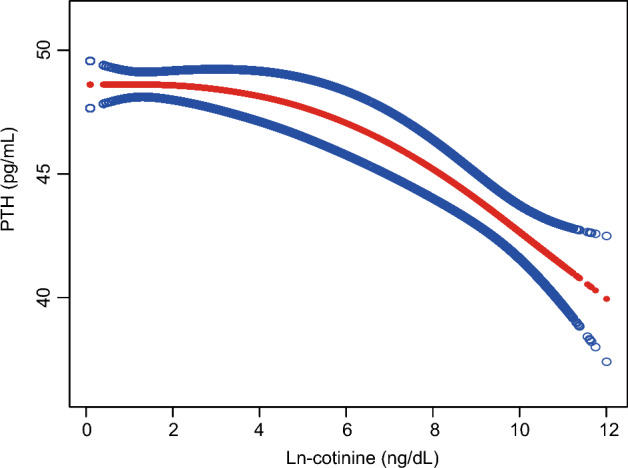
Nonlinear relationship between PTH and cotinine. The red line represents the relationship between PTH and cotinine, while the blue line represents the 95% confidence interval.

In order to establish the nonlinear relationship between cotinine and PTH, a fully adjusted smooth curve was fitted using a generalized additive model (GAM) (Fig. 2). Sensitivity analysis of the GAM model supported the negative correlation between cotinine and PTH, consistent with the results of the multivariate linear regression model. Furthermore, we conducted an analysis of the threshold effect. Among the participants, the threshold effect value between ln-cotinine and PTH was 8.29 ng/dL. After comprehensive adjustment, the impact value β on the left side of the threshold was -0.25 (-0.51, 0.01, p = 0.0602), and the impact value β was -2.10 (-3.12, -1.08, p < 0.001) on the right side of the threshold. Additionally, there was a significant difference in the impact values on both sides of the inflection point, with a p-value less than 0.001 in the likelihood ratio test (Table 3).

**Table 3:** Piecewise linear regression model between serum cotinine and PTH.

Ln-cotinine(ng/dL)	Adjust OR (95% CI) P value
Fitting by linear regression model	−0.58 (−0.72, −0.45) < 0.001*
Fitting by two-piecewise linear regression model	
Inflection point	8.29
< 8.29	−0.25 (−0.51, 0.01) 0.0602
> 8.29	−2.10 (−3.12, −1.08) < 0.001*
Log likelihood ratio test	< 0.001*

*p < 0.05.

## Discussion

Our study is one of the most extensive cross-sectional investigations exploring the potential association between serum cotinine and PTH levels. It is also the first study to investigate this relationship based on data from the NHANES database. Study population consisted of 7641 participants selected from the NHANES 2003–2006. After adjusting for sociodemographic, laboratory, medical examination, personal history, and comorbidity data, we observed a negative correlation between serum cotinine and PTH. Additionally, to assess the non-linear relationship, a smooth curve was fitted, and threshold effect analysis was conducted.

Previous studies on the relationship between tobacco smoke exposure and parathyroid function have yielded conflicting conclusions. A study conducted in 2000 with 489 elderly women indicated elevated PTH levels in heavy smokers^21^, while another study in 2006 involving 74 healthy individuals found reduced PTH levels in smokers^22^. In a study conducted in 2010 involving 1,288 young women and a cross-sectional study in 2016 with 376 young individuals, the frequency of smoking showed a consistent negative correlation with PTH levels^23,24^ which is consistent with our findings. However, two animal experiments regarding nicotine exposure yielded results indicating that stable nicotine exposure does not affect the PTH levels in rats^25,26^. Due to the presence of numerous toxic substances in commercially available cigarettes, this might suggest that the negative correlation between smoke exposure and PTH is not caused by nicotine but rather due to some unknown toxins generated during the smoking process affecting parathyroid function.

Several studies have already pointed out the potential increase in fracture risk and decrease in bone density due to smoke exposure^27,28^. However, PTH does not play an intermediate role in this process, as smoke exposure leads to a decrease in PTH levels, which does not result in a decrease in bone density. Lu, Y. et al. found that smoke exposure impairs the bone remodeling process^29^, but they did not specifically identify the specific harmful components in smoke exposure. Lima, L. L. et al. found that the application of recombinant human parathyroid hormone (PTH 1–34) can mitigate the impact of cigarette smoke on the bone around titanium implants in the oral cavity^30^. A study conducted in 2002 on 405 postmenopausal women indicated that impaired calcium absorption in smokers is largely attributed to the suppression of the PTH-calcitriol endocrine axis^31^. At the same time, in our study, we found an abnormal negative correlation between serum corrected calcium and PTH levels, which further supports this viewpoint.

The lack of statistically significant correlation between cotinine and parathyroid hormone in the subgroup with eGFR less than 60 ml/min/1.73 m^2^ is indeed noteworthy. This discovery suggests a potential difference between tobacco smoke exposure based on renal function and PTH levels. In patients with chronic kidney disease, due to impaired kidney function and imbalanced calcium and phosphorus metabolism, the parathyroid gland is regulated by various complex factors, including renal bone disease and secondary hyperparathyroidism^32^. These factors make the parathyroid function of patients with chronic kidney disease different from that of healthy individuals. In this case, smoking may not have the same effect on PTH levels, and may even have the opposite effect. A study on 161 patients with end-stage renal disease (ESRD) showed that smoking increases the risk of hyperparathyroidism, which is opposite to the trend in our patients with normal kidney function^33^.

Many countries are progressively implementing smoke-free policies to regulate smoking in enclosed public spaces and workplaces. Since the 1990s, following the introduction of tobacco control policies in several states in the United States, smoke-free air ordinances covering public areas (including workplaces, restaurants, and bars) have reached a higher proportion of the U.S. population, increasing from 3.8% to 68.6%^34^. By 2016, a total of 29 states and the District of Columbia had enacted statewide bans on smoking in all public places and workplaces, including bars, restaurants, and private worksites^35^. Against the backdrop of the gradual reinforcement of smoke-free initiatives in public places, exposure to environmental tobacco smoke (ETS) and the corresponding number of cancer-related deaths have also significantly decreased^36,37^. Therefore, it is imperative to expand public health interventions globally to reduce exposure to secondhand smoke (SHS) for cancer prevention and health protection.

Overall, in this large-scale cross-sectional study, after adjusting for confounding factors, a significant negative correlation was observed between serum PTH levels and serum cotinine. However, further exploration may be needed to understand the complex relationship and mechanisms between these two factors. This study has several strengths. Firstly, because cotinine levels in the human body are relatively stable, circulating cotinine can accurately reflect patients’ exposure to smoke, avoiding recall bias caused by questionnaires. Secondly, this is the largest sample study to date regarding the relationship between serum cotinine and PTH. Thirdly, we adjusted for variables that could influence the results and conducted subgroup analyses in our study, making our conclusions about the relationship between the two factors more reliable.

Our study is not without limitations. Firstly, NHANES data is sampled from across the United States, thus study conclusions can be extrapolated to the U.S. population, but may not fully represent the global population. Furthermore, unable to obtain comprehensive and accurate information about the patient's calcium and vitamin D supplementation, as well as the patient's history of parathyroid related diseases. Finally, due to the limitations of cross-sectional studies, it is challenging to follow up with patients, necessitating further large-scale prospective studies to confirm the conclusions.

## Conclusions

This study reveals a negative correlation between serum cotinine levels and PTH levels, implying that tobacco smoke exposure may lead to parathyroid dysfunction in adults. Future research should concentrate on exploring the association and underlying mechanisms between tobacco smoke exposure and the body's calcium-phosphate balance and parathyroid function. Such research endeavors hold promise for advancing public policies aimed at fostering smoke-free environments.

### Supplementary Information

Supplementary Information.

## Data Availability

The data used in the present study are all publicly available at https://www.cdc.gov/nchs/nhanes/index.htm.
