# Supplementary material for: Association between tobacco smoke exposure and serum parathyroid hormone levels among US adults (NHANES 2003–2006)
Source: Sci Rep. 2024 Jul 9;14:15781. doi: 10.1038/s41598-024-66937-7 (PMC11233680; doi:10.1038/s41598-024-66937-7)
Supplement: Supplementary file 1 — Supplementary Information. [file 41598_2024_66937_MOESM1_ESM.docx]

**Association between tobacco smoke exposure and serum parathyroid hormone levels among US adults (NHANES 2003-2006)**

**Longqing Hu M.D. ^1, #^, Bei Qian M.D. ^1, #^, Kaijian Bing M.D. ^1^, Li Mei M.D. ^1^, Shengnan Ruan M.D. ^1, *^, Xincai Qu M.D. & Ph.D^1, *^.**

**Supplementary Table**

Supplementary Table S1 Participant baseline characteristics

| **PTH (pg/mL)**  **41.00 (31.00, 51.00)** | **Q1**  **(n=1842)** | **Q2**  **(n=1871)** | **Q3**  **(n=1940)** | **Q4**  **(n=1988)** | **P value** |
| --- | --- | --- | --- | --- | --- |
| **Cycle** |  |  |  |  | 0.2349 |
| 2003-2004 | 51.46 | 47.65 | 49.31 | 50.04 |  |
| 2005-2006 | 48.54 | 52.35 | 50.69 | 49.96 |  |
| **Gender** |  |  |  |  | <0.001^*^ |
| Male | 53.40 | 53.40 | 47.14 | 45.27 |  |
| Female | 46.60 | 46.60 | 52.86 | 54.73 |  |
| **Race** |  |  |  |  | <0.001^*^ |
| Mexican American | 6.02 | 8.47 | 8.96 | 10.00 |  |
| Other Hispanic | 3.07 | 3.81 | 4.36 | 3.84 |  |
| Non-Hispanic White | 77.20 | 72.56 | 70.16 | 62.81 |  |
| Non-Hispanic Black | 8.91 | 9.50 | 11.15 | 17.25 |  |
| Other Race | 4.79 | 5.65 | 5.36 | 6.11 |  |
| **Education** |  |  |  |  | 0.0024^*^ |
| Lower than High school | 15.90 | 15.64 | 18.97 | 21.13 |  |
| High school | 26.84 | 26.2 | 25.19 | 25.51 |  |
| College or above | 57.26 | 57.26 | 55.83 | 53.36 |  |
| **Diabetes** |  |  |  |  | 0.0870 |
| Yes | 6.89 | 6.93 | 6.84 | 9.01 |  |
| No | 92.24 | 91.9 | 91.6 | 89.42 |  |
| Borderline | 0.87 | 1.17 | 1.56 | 1.57 |  |
| **Hypertension** |  |  |  |  | <0.001^*^ |
| Yes | 22.59 | 23.93 | 30.05 | 40.68 |  |
| No | 77.41 | 76.07 | 69.95 | 59.32 |  |
| **Alcohol use** |  |  |  |  | <0.001^*^ |
| Yes | 81.18 | 75.11 | 71.57 | 64.17 |  |
| No | 18.82 | 24.89 | 28.43 | 35.83 |  |
| **Smoke** |  |  |  |  | <0.001^*^ |
| Yes | 56.28 | 50.03 | 47.37 | 42.50 |  |
| No | 43.72 | 49.97 | 52.63 | 57.50 |  |
| **CKD** |  |  |  |  | <0.001^*^ |
| Yes | 3.03 | 3.75 | 6.46 | 15.02 |  |
| No | 96.97 | 96.25 | 93.54 | 84.98 |  |
| **Age (year)** | 39.00 (28.00, 50.00) | 44.00 (32.00, 54.00) | 46.00 (35.00, 58.00) | 50.00 (37.00, 64.00) | <0.001^*^ |
| **Family PIR** | 3.10 (1.65, 4.89) | 3.20 (1.68, 5.00) | 3.11 (1.61, 4.83) | 2.73 (1.44, 4.55) | <0.001^*^ |
| **Cotinine(ng/dL)** | 55.90 (3.20, 20100.00) | 8.30 (2.20, 4620.00) | 7.00 (2.10, 349.00) | 6.00 (2.00, 106.00) | <0.001^*^ |
| **BMI (kg/m^2^)** | 25.97 (22.99, 29.98) | 27.08 (23.90, 31.18) | 27.93 (24.24, 32.08) | 29.31 (25.36, 34.20) | <0.001^*^ |
| **25 (OH) D_3_ (nmol/L)** | 66.50 (53.20, 82.70) | 61.60 (48.30, 76.20) | 58.10 (43.40, 71.30) | 50.80 (36.00, 65.50) | <0.001^*^ |
| **Serum albumin (g/dL)** | 4.30 (4.10, 4.50) | 4.30 (4.10, 4.50) | 4.30 (4.10, 4.50) | 4.20 (4.00, 4.40) | <0.001^*^ |
| **Serum creatinine (mg/dL)** | 0.90 (0.80, 1.00) | 0.90 (0.80, 1.00) | 0.90 (0.80, 1.00) | 0.90 (0.80, 1.10) | <0.001^*^ |
| **ALP (U/L)** | 62.00 (51.00, 76.00) | 64.00 (53.00, 77.00) | 67.00 (55.00, 80.00) | 70.00 (58.00, 85.00) | <0.001^*^ |
| **Corrected-Ca (mg/dL)** | 9.60 (9.40, 9.80) | 9.58 (9.38, 9.80) | 9.50 (9.30, 9.70) | 9.48 (9.26, 9.70) | <0.001^*^ |
| **Cholesterol (mg/dL)** | 196.00 (172.00, 225.00) | 198.00 (174.00, 227.00) | 199.00 (173.00, 230.00) | 198.00 (172.00, 227.00) | 0.4436 |
| **Triglycerides (mg/dL)** | 111.00 (72.00, 174.00) | 110.00 (75.00, 174.00) | 114.00 (76.00, 182.00) | 117.00 (81.00, 174.00) | 0.0522 |
| **eGFR (ml/min/1.73 m^2^)** | 99.24 (85.63, 111.83) | 95.88 (81.21, 109.57) | 93.16 (78.41, 107.21) | 88.08 (70.69, 105.72) | <0.001^*^ |
| **Serum glucose (mg/dL)** | 89.00 (82.00, 97.00) | 90.00 (84.00, 99.00) | 92.00 (85.00, 99.00) | 92.00 (86.00, 101.00) | <0.001^*^ |
| **Serum phosphorus (mg/dL)** | 3.80 (3.50, 4.20) | 3.80 (3.50, 4.10) | 3.70 (3.40, 4.10) | 3.70 (3.40, 4.00) | <0.001^*^ |
| **Urinary albumin (ug/mL)** | 31.00 (6.60, 117.00) | 27.00 (6.70, 114.00) | 33.00 (7.80, 126.00) | 45.00 (8.90, 137.00) | <0.001^*^ |
| **Urinary creatinine (mg/dL)** | 25.70 (5.70, 114.00) | 37.00 (6.50, 123.00) | 36.00 (7.50, 125.00) | 43.00 (8.80, 125.00) | <0.001^*^ |

PIR, poverty income ratio; BMI, body mass index; ALP, alkaline phosphatase; ^*^, p<0.05.

**Figure legends**

Figure 1 Participant inclusion flowchart

Figure 2 Nonlinear relationship between PTH and cotinine

The red line represents the relationship between PTH and cotinine, while the blue line represents the 95% confidence interval.
